# Comparative Protective Effects of Melatonin and Apigenin Against Paclitaxel-Induced Testicular Injury in Rats: Oxidative Stress, DNA Damage, Apoptosis, and NF-κB Signaling

**DOI:** 10.3390/nu18101643

**Published:** 2026-05-21

**Authors:** Faruk Saydam, Merve Altuntoprak, Enes Bahadir Bay, Tolga Mercantepe, Atilla Topcu, Sibel Mataraci Karakas

**Affiliations:** 1 Department of Medical Biology, Faculty of Medicine, Recep Tayyip Erdogan University, 53100 Rize, Türkiye; faruk.saydam@erdogan.edu.tr; 2 Faculty of Medicine, Recep Tayyip Erdogan University, 53100 Rize, Türkiye; merve_altuntoprak22@erdogan.edu.tr (M.A.); enesbahadir_bay22@erdogan.edu.tr (E.B.B.); 3 Department of Histology and Embryology, Faculty of Medicine, Recep Tayyip Erdogan University, 53100 Rize, Türkiye; tolga.mercantepe@erdogan.edu.tr; 4 Department of Pharmacology, Faculty of Medicine, Recep Tayyip Erdogan University, 53100 Rize, Türkiye; atilla.topcu@erdogan.edu.tr; 5 Department of Medical Biochemistry, Faculty of Medicine, Recep Tayyip Erdogan University, 53100 Rize, Türkiye

**Keywords:** apigenin, chemotherapy-induced toxicity, infertility, melatonin, paclitaxel

## Abstract

Background: Paclitaxel is a widely used chemotherapeutic agent whose clinical efficacy is limited by gonadotoxic side effects. Oxidative stress, apoptosis, and inflammation are key mechanisms underlying paclitaxel-induced testicular injury. This study aimed to comparatively evaluate the protective effects of melatonin and apigenin in a rat model. Methods: Adult male Sprague-Dawley rats were randomly assigned to seven groups: control, solvent control, melatonin, apigenin, paclitaxel, paclitaxel + melatonin, and paclitaxel + apigenin. Testicular malondialdehyde (MDA) and reduced glutathione (GSH) levels were measured, together with apoptotic activity (caspase-3), oxidative DNA damage (8-OHdG), inflammatory signaling (NF-κB/p65, immunoreactivity), and histopathological alterations (Johnsen score). Results: Paclitaxel significantly increased MDA levels and decreased GSH content, accompanied by elevated caspase-3, 8-OHdG, and NF-κB/p65 immunoreactivity, as well as marked degeneration of seminiferous tubules. Melatonin improved redox balance, suppressed apoptotic and inflammatory responses, and preserved testicular architecture. Apigenin reduced lipid peroxidation and improved antioxidant status in paclitaxel-treated rats while decreasing GSH levels under basal conditions without inducing histological damage, suggesting a context-dependent redox-modulating effect. Both agents significantly improved Johnsen scores compared with paclitaxel alone. Conclusions: Paclitaxel-induced testicular injury is mediated by a coordinated interplay of oxidative stress, apoptosis, inflammation, and structural degeneration. Melatonin and apigenin effectively mitigate these processes, with apigenin exhibiting context-dependent antioxidant activity. These findings suggest that melatonin and apigenin may serve as adjunctive strategies for preserving male reproductive function during chemotherapy.

## 1. Introduction

Cancer remains a major global health burden, with approximately 20 million new cases and nearly 10 million deaths reported worldwide in 2022 [1]. Systemic chemotherapy plays a central role in cancer treatment; however, its clinical efficacy is often limited by adverse effects on non-target tissues, including the male reproductive system [2,3]. Among these, impairment of spermatogenesis represents a clinically significant but often underestimated complication.

Paclitaxel, a widely used chemotherapeutic agent in the treatment of solid tumors such as breast, ovarian, and lung cancers, has been shown to induce testicular toxicity [4,5,6,7,8]. Experimental evidence indicates that paclitaxel disrupts the blood–testis barrier, induces DNA damage, and impairs germ cell integrity, ultimately leading to structural degeneration of seminiferous tubules and reduced fertility potential [9,10].

Disruption of redox homeostasis is a key mechanism underlying chemotherapy-induced gonadal toxicity. Paclitaxel promotes an imbalance between reactive oxygen species (ROS) production and antioxidant defense systems, resulting in lipid peroxidation and cellular damage [11,12]. Malondialdehyde (MDA) is a widely used marker of lipid peroxidation, whereas reduced glutathione (GSH) plays a critical role in maintaining intracellular redox balance [13,14]. This imbalance activates apoptotic signaling pathways, including caspase-3, and triggers inflammatory cascades, with nuclear factor kappa B (NF-κB) acting as a central regulator linking oxidative stress to inflammation and apoptosis [15,16,17,18]. In addition, oxidative DNA damage markers such as 8-hydroxy-2′-deoxyguanosine (8-OHdG) reflect ROS-mediated genomic injury and are associated with impaired spermatogenesis [6].

Given the multifactorial nature of paclitaxel-induced testicular injury, there is growing interest in identifying adjunctive agents capable of modulating oxidative stress, apoptosis, and inflammation. Melatonin, an endogenous indoleamine, exhibits potent antioxidant and anti-inflammatory properties and has been shown to mitigate chemotherapeutic-induced reproductive toxicity [19,20,21,22,23]. Apigenin, a naturally occurring flavonoid, also demonstrates antioxidant, anti-inflammatory, and anti-apoptotic effects and has been reported to protect against testicular damage in experimental models [24,25,26].

Despite evidence supporting the individual protective effects of melatonin and apigenin, direct comparative studies evaluating their efficacy and underlying mechanisms under identical experimental conditions remain limited. To our knowledge, this is the first study to systematically compare these two bioactive compounds in a paclitaxel-induced testicular toxicity model.

Therefore, the present study aimed to comparatively evaluate the protective effects of melatonin and apigenin in a rat model of paclitaxel-induced testicular injury. Oxidative stress markers (MDA and GSH), apoptotic activity (caspase-3), oxidative DNA damage (8-OHdG), inflammatory signaling (NF-κB/p65), and histopathological alterations assessed by Johnsen scoring were evaluated to elucidate the mechanistic basis of paclitaxel-induced gonad toxicity and to identify potential therapeutic strategies for preserving testicular integrity and spermatogenic structure during chemotherapy.

## 2. Materials and Methods

### 2.1. Chemicals

Sodium chloride (NaCl) was obtained from Biofarma İlaç Sanayi ve Ticaret A.Ş. (Istanbul, Türkiye). Paclitaxel (Taksen^®^, 300 mg/50 mL, dissolved in 0.9% saline) was supplied by Koçak İlaç Sanayi (Istanbul, Türkiye). Anesthesia was induced using ketamine hydrochloride (Ketalar^®^, Eczacıbaşı Parke-Davis, Istanbul, Türkiye) and xylazine hydrochloride (Alfazyne^®^, Alfasan International B.V., Woerden, The Netherlands). All other chemicals and reagents used in the study were of analytical grade and purchased from Sigma-Aldrich (St. Louis, MO, USA) and Merck (Darmstadt, Germany).

### 2.2. Animals and Experimental Design

All experimental animal procedures were performed in accordance with the ARRIVE 2.0 guidelines describing animal research [27]. Adult male Sprague Dawley rats (weighing 260 ± 31 g) were obtained from the Experimental Animal Research Center of Recep Tayyip Erdoğan University. Animals were housed under controlled laboratory conditions (24 ± 2 °C, 45 ± 5% relative humidity, and a 12 h light/dark cycle) with free access to standard pellet chow and water *ad libitum*. All experimental procedures were conducted in accordance with the National Institutes of Health Guide for the Care and Use of Laboratory Animals and were approved by the Institutional Animal Ethics Committee (Decision number: 2023/49).

Rats were randomly assigned to seven equal groups (*n* = 8 per group) as follows:

- **Control (SF, Saline, Group 1)**: Intraperitoneal administration of 1 mL 0.9% saline (SF) once weekly for 4 weeks.
- **Solvent control (DMSO, Dimethyl Sulfoxide, Group 2)**: Intraperitoneal administration of 1 mL 1% DMSO once weekly for 4 weeks.
- **Melatonin (Group 3)**: Melatonin (10 mg/kg) was dissolved in saline containing 1% (*v*/*v*) ethanol, resulting in a final ethanol concentration ≤1% (*v*/*v*), and administered intraperitoneally once weekly for 4 weeks.
- **Apigenin (Group 4)**: Apigenin (27 mg/kg) dissolved in DMSO, administered intraperitoneally once weekly for 4 weeks.
- **Paclitaxel (Group 5)**: Paclitaxel (5 mg/kg) dissolved in 0.9% NaCl and administered intraperitoneally once weekly for 4 weeks.
- **Paclitaxel + Melatonin (Group 6)**: Melatonin (10 mg/kg) was administered 30 min before paclitaxel (5 mg/kg) administration, intraperitoneally once weekly for 4 weeks.
- **Paclitaxel + Apigenin (Group 7)**: Apigenin (27 mg/kg) was administered 30 min before paclitaxel (5 mg/kg) administration, intraperitoneally once weekly for 4 weeks.

The final concentration of DMSO in all apigenin-treated groups was identical to that used in the solvent control group. Melatonin was prepared in saline containing 1% (*v*/*v*) ethanol, a solvent widely used for intraperitoneal melatonin administration in rodent models. Although a separate ethanol control group was not included, previous studies have demonstrated that intraperitoneal administration of ethanol at concentrations ≤1% (*v*/*v*) does not induce oxidative stress or histological alterations in rat testicular tissue [23,25,26]. Solvent concentrations were kept low and within commonly used inert ranges (DMSO 1%; ethanol ≤ 1% *v*/*v*).

Melatonin (10 mg/kg) and apigenin (27 mg/kg) doses were selected based on previous experimental studies demonstrating their antioxidant and cytoprotective efficacy in rodent models of oxidative and inflammatory tissue injury. The selected apigenin dose falls within the effective range (10–50 mg/kg) reported in the literature and was chosen as an intermediate dose to achieve robust biological effects while minimizing the risk of dose-related variability or potential adverse effects [3,23,28].

At the end of the experimental period, animals were anesthetized using ketamine hydrochloride (100 mg/kg, i.p.) and xylazine hydrochloride (10 mg/kg, i.p.) and sacrificed by cervical dislocation. The testes were removed, weighed, and prepared for biochemical, histopathological and immunohistochemical analyses.

### 2.3. Tissue Collection and Preparation

Immediately after excision, the testicular tissues were washed with ice-cold saline to eliminate residual blood. Samples intended for biochemical analysis were snap-frozen and stored at −80 °C until further processing. Tissues allocated for histological and immunohistochemical examination were fixed in 10% neutral buffered formalin.

### 2.4. Biochemical Analysis

#### 2.4.1. Preparation of Tissue Homogenates

Before homogenization, frozen tissues were thawed on ice to minimize enzymatic degradation. Each sample was weighed and homogenized in cold phosphate buffer (20 mM sodium phosphate, 140 mM potassium chloride, pH 7.4) at a ratio of 1:9 (*w*/*v*). Homogenization was performed using a TissueLyser II system (QIAGEN, Hilden, Germany) at 30 Hz for 5 min. Following homogenization, samples were centrifuged at 800× *g* for 10 min at 4 °C. The resulting supernatants were carefully collected and used for biochemical analyses.

#### 2.4.2. Measurement of Malondialdehyde (MDA)

Lipid peroxidation was quantified by measuring MDA formation using a thiobarbituric acid–based assay. Briefly, tissue supernatants were incubated with thiobarbituric acid under acidic and high-temperature conditions to allow formation of a chromogenic adduct. Following incubation, absorbance was measured at 532 nm using a spectrophotometer. MDA concentrations were determined from a standard curve and normalized to tissue weight. Results were expressed as nmol per gram of tissue [13].

#### 2.4.3. Determination of Reduced Glutathione (GSH)

GSH levels were determined based on its reaction with 5,5′-dithiobis (2-nitrobenzoic acid) (DTNB), yielding a colored thiol-DTNB complex. Absorbance was recorded at 412 nm, and GSH concentrations were calculated using a calibration curve generated from known standards. Measured values were converted to total GSH content according to homogenate volume and normalized to tissue weight. Data were expressed as µmol per gram of tissue [14].

All analyses for MDA and GSH were repeated three times; blank values were excluded.

### 2.5. Histopathological Analysis

Formalin-fixed testicular tissues were processed using standard histological procedures, embedded in paraffin, and sectioned at 4–5 µm thickness. Sections were stained with hematoxylin and eosin (H&E) and examined under a light microscope.

Spermatogenic activity was evaluated using the Johnsen testicular biopsy score. Fifty seminiferous tubules were randomly selected from different regions in each group and scored accordingly (Table 1) [29]. An expert histopathologist blinded to the study groups conducted the scoring by examining 20 different areas of each rat testicle. Scores ranged from 1 (no germ cells) to 10 (complete spermatogenesis) [29]. Evaluations were performed by an experienced histologist to ensure consistency.

### 2.6. Immunohistochemical (IHC) Analysis

Immunohistochemical analysis was performed to evaluate apoptotic activity, oxidative DNA damage, and inflammatory signaling in testicular tissue. Sections were immunostained for active caspase-3 (1/100, ab4051, Abcam, Cambridge, UK), 8-hydroxy-2′-deoxyguanosine (8-OHdG; 1/200, sc-66036, Santa Cruz Biotechnology, Inc., Dallas, TX, USA), and NF-κB/p65 (1/200, BT-AP05991, Shanghai Korain Biotech, Shanghai, China). Following fixation in neutral buffered formalin and routine paraffin embedding, 4 µm tissue sections were mounted on positively charged slides (PatoLab, Istanbul, Türkiye). Antigen retrieval was conducted using citrate buffer (pH 6.0).

Immunostaining was performed using an automated staining system according to the manufacturer’s protocol. After incubation with primary antibodies, signal detection was achieved using a 3,3-diaminobenzidine (DAB) chromogen, followed by hematoxylin counterstaining. Negative controls were processed in parallel with omission of the primary antibody.

Immunoreactivity was evaluated semi-quantitatively in a blinded manner. For each animal, five sections were examined and 10 non-overlapping fields per section were evaluated at ×400 magnification. Immunopositivity was graded using a 0–3 scoring system based on overall staining extent/intensity: 0 = no immunoreactivity; 1 = weak/limited immunoreactivity; 2 = moderate immunoreactivity; 3 = strong/widespread immunoreactivity. Scores were independently assessed by two observers, and discrepancies were resolved by joint review.

### 2.7. Statistical Analysis

All statistical analyses were performed using SPSS software (version 18.0). Data were tested for normality using the Shapiro–Wilk test. Parametric data were expressed as mean ± standard error of the mean (SEM) and analyzed using one-way analysis of variance (ANOVA) followed by the LSD post hoc test. The LSD post hoc test was used for planned pairwise comparisons due to the limited number of experimental groups. Non-parametric data were expressed as median (interquartile range) and analyzed using the Kruskal–Wallis test followed by the Dunn test for pairwise comparisons. *p* < 0.05 was considered statistically significant.

## 3. Results

### 3.1. Biochemical Results

MDA and GSH data exhibited normal distribution (*p* > 0.05). Significant differences were found between the experimental groups in terms of MDA and GSH levels (*p* = 0.001) (Table 2).

MDA levels were significantly increased in the paclitaxel-treated group compared with the SF, apigenin, and melatonin groups (*p* = 0.006, *p* = 0.031, and *p* = 0.003, respectively), indicating enhanced lipid peroxidation following paclitaxel administration. Co-administration of melatonin with paclitaxel resulted in a significant reduction in MDA levels compared with paclitaxel alone (*p* = 0.001). Given the comparable MDA values in the DMSO and paclitaxel groups, SF served as the primary comparator for interpreting paclitaxel-associated lipid peroxidation. Importantly, MDA levels were significantly lower in the paclitaxel + apigenin group than in the DMSO group (*p* = 0.001), supporting attenuation of lipid peroxidation in paclitaxel-exposed rats following apigenin treatment.

GSH levels were significantly decreased in the paclitaxel group compared with the SF group (*p* < 0.001). Co-administration of melatonin with paclitaxel resulted in a significant increase in GSH levels compared with paclitaxel alone (*p* < 0.001). When DMSO was used as the solvent control, significant differences in GSH levels were observed between the DMSO group and the paclitaxel, apigenin, and paclitaxel + apigenin groups (*p* < 0.001 for all comparisons). Notably, apigenin administration alone resulted in a significant reduction in GSH levels compared with the DMSO group (*p* < 0.001). However, in paclitaxel-treated rats, apigenin co-administration significantly increased GSH levels compared with the paclitaxel group (*p* = 0.005).

This decrease in GSH levels in the apigenin-alone group, despite the absence of histopathological damage, suggests a context-dependent modulation of redox homeostasis rather than overt oxidative stress.

### 3.2. Histopathological Findings

**Figure 1:**
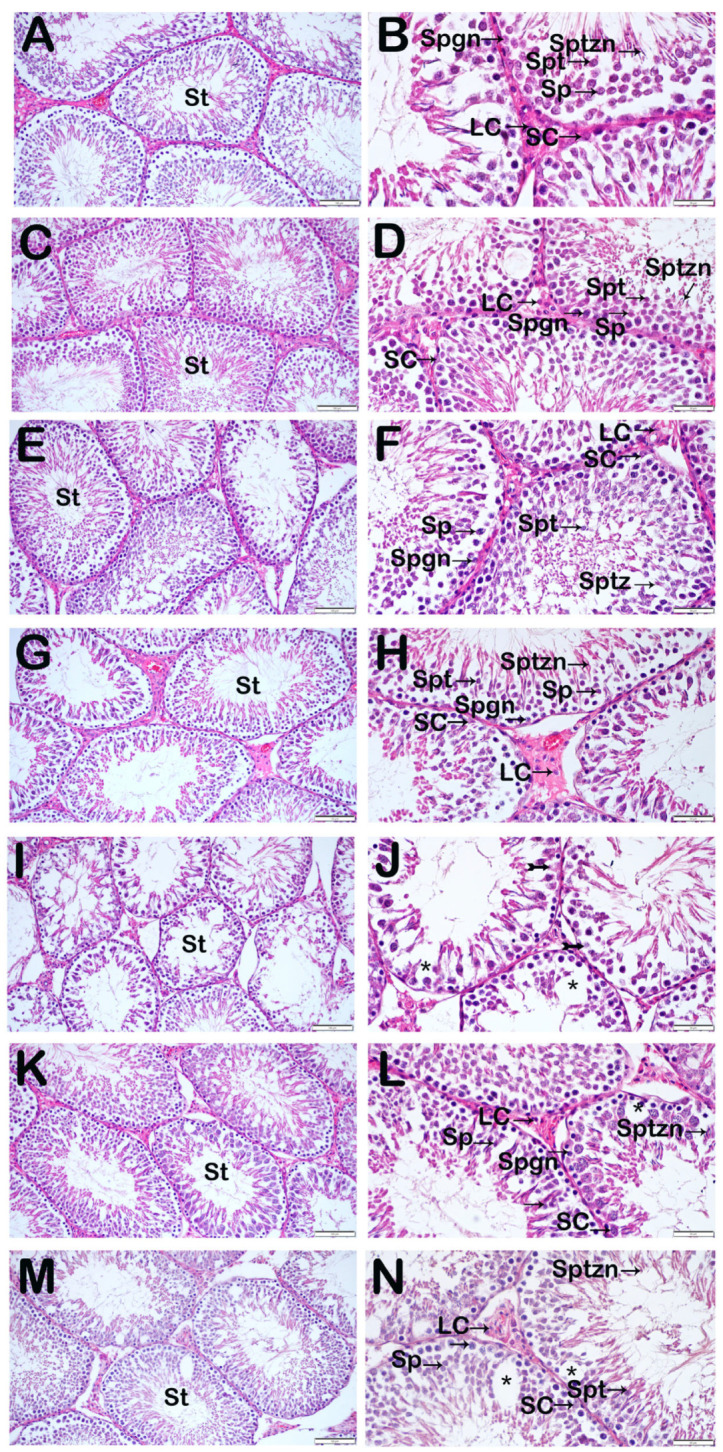
Representative light microscopic image of sections of testicular tissue stained with H&E. Seminiferous tubules (St), Spermatogonia (Spgn), Primary spermatocyte (Sp), Spermatid (Spt), Spermatozoa (Sptzn), Sertoli cell (SC), Leydig cells (LC), Edematous area (*). (**A**) (×20)–(**B**) (×40) DMSO group: Testicular tissue showed normal seminiferous tubule architecture with intact spermatogenic cells and interstitial structures (Johnsen’s score: 9 (9–9)). (**C**) (×20)–(**D**) (×40) SF group: Seminiferous tubules displayed typical morphology with preserved germinal epithelium (Johnsen’s score: 9 (8–9)). (**E**) (×20)–(**F**) (×40) Apigenin group: Normal testicular histology with well-organized seminiferous tubules and intact spermatogenic cells (Johnsen’s score: 9 (8–9)). (**G**) (×20)–(**H**) (×40) Melatonin group: Seminiferous tubules showed preserved germinal epithelium and normal cellular organization (Johnsen’s score: 9 (8–9)). (**I**) (×20)–(**J**) (×40) Paclitaxel group: Marked degeneration characterized by edema (star) and loss of spermatogenic cells, particularly spermatids and spermatozoa (spiral arrow) (Johnsen’s score: 5 (4–6)). (**K**) (×20)–(**L**) (×40) Paclitaxel + Apigenin group: Seminiferous tubules showed preserved spermatogenic structure (Johnsen’s score: 8 (8–9)). (**M**) (×20)–(**N**) (×40) Paclitaxel + Melatonin group: Reduced spermatogenic cell loss with partial structural recovery (Johnsen’s score: 8 (7–8)).

Testicular tissue sections stained with hematoxylin and eosin demonstrated normal seminiferous tubule architecture with intact spermatogenic cells and interstitial Leydig cells in the DMSO group (Table 3, Figure 1A,B; Johnsen’s score: 9 (9–9)). Comparable findings were observed in the SF group, with preserved germinal epithelium and normal terstitial cell morphology. The apigenin and melatonin groups exhibited normal histological features, including well-organized seminiferous tubules and intact spermatogenic cell populations (Table 3, Figure 1E–H; Johnsen’s score: 9 (8–9) for both groups). In contrast, the paclitaxel group showed marked degeneration characterized by interstitial edema and loss of spermatogenic cells, particularly spermatids and spermatozoa (Table 3, Figure 1I,J; Johnsen’s score: 5 (4–6)). Both combination treatment groups demonstrated partial histological recovery. The paclitaxel + apigenin group exhibited reduced edema and decreased spermatogenic cell loss, with partial preservation of seminiferous tubule architecture (Table 3, Figure 1K,L; Johnsen’s score: 8 (8–9)). Similarly, the paclitaxel + melatonin group showed reduced edema and improved preservation of spermatogenic cells (Table 3, Figure 1M,N; Johnsen’s score: 8 (7–8)).

### 3.3. Immunohistochemical (IHC) Findings

Quantitative immunohistochemical analysis revealed a consistent pattern across all three markers (active caspase-3, 8-OHdG, and NF-κB/p65). Paclitaxel administration markedly increased immunoreactivity, whereas melatonin and apigenin treatments significantly attenuated these elevations. No relevant immunopositivity was observed in control or treatment-alone groups, indicating the absence of basal apoptotic, oxidative, or inflammatory activation. Immunohistochemical positivity scores for active caspase-3, 8-OHdG and NF-κB/p65 are presented in Table 4.

#### 3.3.1. Active Caspase-3

**Figure 2:**
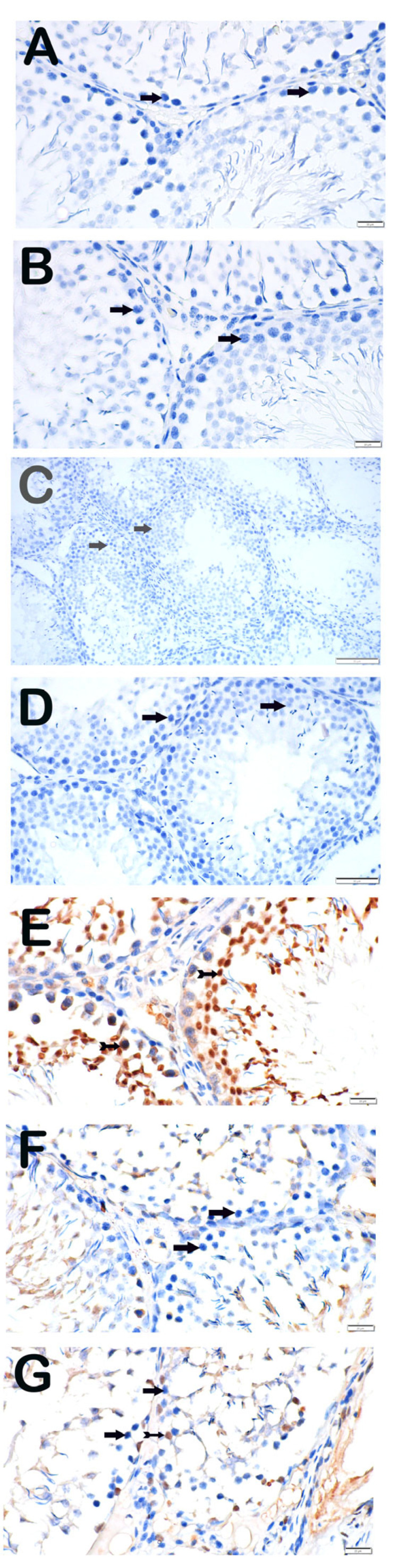
Representative light microscopic image of sections of testicular tissue incubated with the active caspase-3 primary antibody. (**A**) (×40) DMSO group: Spermatogenic cells with normal structure were immunonegative for caspase-3 (arrow; positivity score: 0 (0–0)). (**B**) (×40) SF group: Spermatogenic cells were immunonegative for caspase-3 (arrow; positivity score: 0 (0–0)). (**C**) (×40) Apigenin group: Spermatogenic cells were immunonegative for caspase-3 (arrow; positivity score: 0 (0–0)). (**D**) (×40) Melatonin group: Spermatogenic cells were immunonegative for caspase-3 (arrow; positivity score: 0 (0–0)). (**E**) (×40) Paclitaxel group: Spermatogenic cells showed strong caspase-3 immunopositivity (arrowhead; positivity score: 3 (2–3)). (**F**) (×40) Paclitaxel + Apigenin group: Reduced caspase-3 immunopositivity was observed (tail arrow; positivity score: 1 (1–1)). (**G**) (×40) Paclitaxel + Melatonin group: Reduced caspase-3 immunopositivity was observed (spiral arrow; positivity score: 1 (1–1)).

Caspase-3 immunoreactivity was absent in control and treatment-alone groups. In contrast, the Paclitaxel group exhibited strong positivity (3 (2–3)), which was significantly reduced in both treatment groups (Paclitaxel + Apigenin and Paclitaxel + Melatonin) (1 (1–1)) (Figure 2).

#### 3.3.2. 8-OHdG

**Figure 3:**
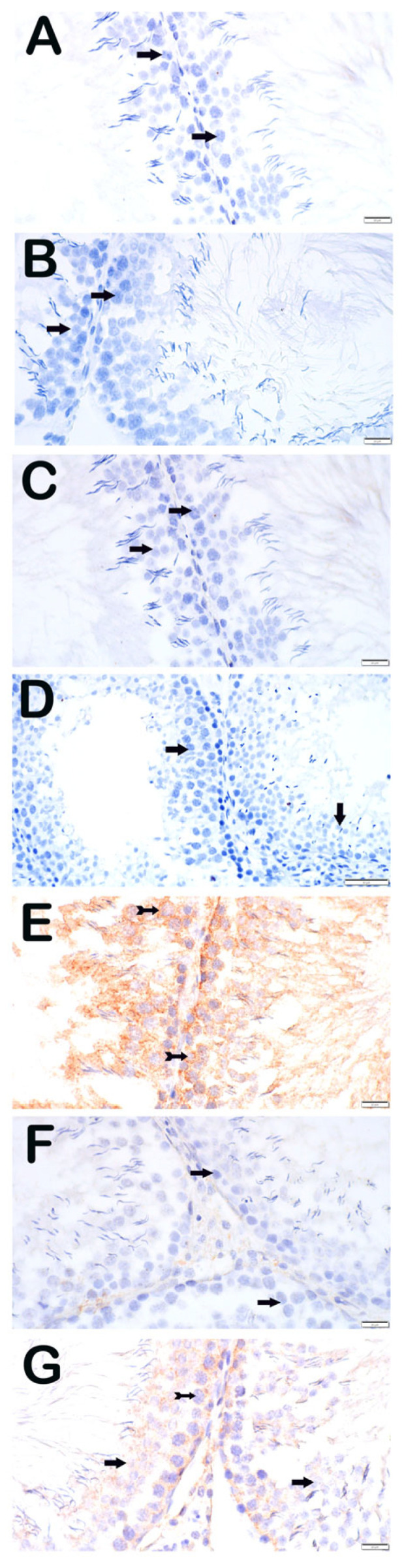
Representative light microscopic image of sections of testicular tissue incubated with the 8-OHdG primary antibody. (**A**) (×40) DMSO group: Spermatogenic cells with normal structure were immunonegative for 8-OHdG (arrow; positivity score: 0 (0–0)). (**B**) (×40) SF group: Spermatogenic cells were immunonegative for 8-OHdG (arrow; positivity score: 0 (0–0)). (**C**) (×40) Apigenin group: Spermatogenic cells were immunonegative for 8-OHdG (arrow; positivity score: 0 (0–0)). (**D**) (×40) Melatonin group: Spermatogenic cells were immunonegative for 8-OHdG (arrow; positivity score: 0 (0–1)). (**E**) (×40) Paclitaxel group: Spermatogenic cells showed strong 8-OHdG immunopositivity (arrowhead; positivity score: 3 (2–3)). (**F**) (×40) Paclitaxel + Apigenin group: Reduced 8-OHdG immunopositivity was observed (tail arrow; positivity score: 1 (1–1)). (**G**) (×40) Paclitaxel + Melatonin group: Reduced 8-OHdG immunopositivity was observed (spiral arrow; positivity score: 1 (1–1)).

Similarly, 8-OHdG immunoreactivity was minimal in control and treatment-alone groups but markedly increased in the Paclitaxel group (3 (2–3)). Both treatment groups (Paclitaxel + Apigenin and Paclitaxel + Melatonin) showed significantly decreased positivity (1 (1–1)) (Figure 3).

#### 3.3.3. NF-κB/p65

**Figure 4:**
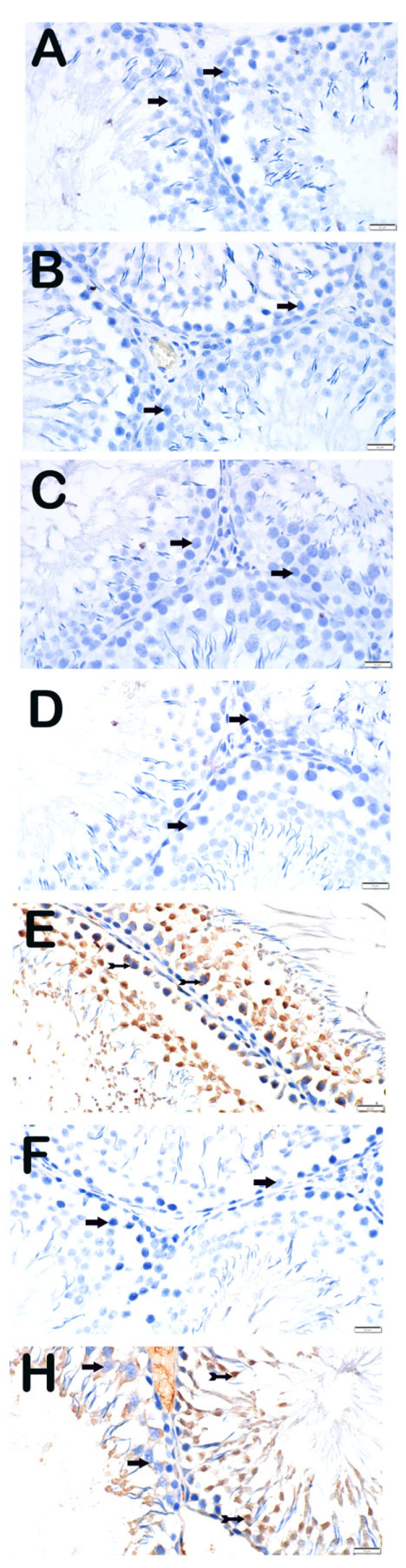
Representative light microscopic image of sections of testicular tissue incubated with the NF-κB/p65 primary antibody. (**A**) (×40) DMSO group: Spermatogenic cells with normal structure were immunonegative for NF-κB/p65 (arrow; positivity score: 0 (0–0)). (**B**) (×40) SF group: Spermatogenic cells were immunonegative for NF-κB/p65 (arrow; positivity score: 0 (0–0)). (**C**) (×40) Apigenin group: Spermatogenic cells were immunonegative for NF-κB/p65 (arrow; positivity score: 0 (0–0)). (**D**) (×40) Melatonin group: Spermatogenic cells were immunonegative for NF-κB/p65 (arrow; positivity score: 0 (0–1)). (**E**) (×40) Paclitaxel group: Spermatogenic cells showed strong NF-κB/p65 immunopositivity (arrowhead; positivity score: 3 (2–3)). (**F**) (×40) Paclitaxel + Apigenin group: Reduced NF-κB/p65 immunopositivity was observed (arrowhead; positivity score: 1 (1–1)). (**H**) (×40) Paclitaxel + Melatonin group: Reduced NF-κB/p65 immunopositivity was observed (spiral arrow; positivity score: 1 (1–1)).

NF-κB/p65 immunoreactivity followed a comparable pattern, with negligible staining in control groups and strong positivity in the Paclitaxel group (3 (2–3)). This increase was effectively attenuated in both treatment groups (Paclitaxel + Apigenin and Paclitaxel + Melatonin), resulting in low to moderate immunoreactivity (0–1) (Figure 4).

## 4. Discussion

Paclitaxel-induced testicular toxicity appears to involve a complex interplay between oxidative stress, inflammatory signaling, and apoptotic activation, ultimately leading to degeneration of seminiferous tubules. In this regard, our findings demonstrate that both melatonin and apigenin substantially attenuate these pathological cascades, supporting their protective effects by restoring redox homeostasis, downregulating inflammatory signaling, and suppressing apoptosis-related pathways.

The concomitant elevation in MDA and reduction in GSH indicate that paclitaxel markedly impairs redox balance in testicular tissue. These findings are consistent with previous studies reporting that taxane-based chemotherapeutic agents promote excessive reactive oxygen species (ROS) generation, leading to lipid peroxidation and antioxidant depletion [11,12]. Given the high polyunsaturated fatty acid content of germ cell membranes, testicular tissue is particularly susceptible to oxidative injury, which can compromise membrane integrity and cellular viability [13].

Oxidative stress-mediated redox imbalance is closely linked to the activation of apoptotic pathways in testicular tissue. NF-κB/p65 immunoreactivity was markedly increased following paclitaxel treatment, indicating activation of pro-inflammatory transcriptional pathways. NF-κB is known to be activated by oxidative stress and, in turn, amplifies tissue damage by upregulating inflammatory mediators and pro-apoptotic genes [17]. Consistent with previous studies, enhanced NF-κB signaling was accompanied by increased caspase-3 activation and oxidative DNA damage [18]. The parallel increase in NF-κB/p65, caspase-3, and 8-OHdG suggests that inflammatory signaling, apoptosis, and oxidative DNA damage may be interconnected processes in paclitaxel-induced testicular injury. However, these findings should be interpreted with caution, as they are correlative in nature and do not establish direct causal relationships between these pathways. Further studies employing targeted pathway inhibition or molecular interventions are required to elucidate the underlying mechanisms.

Melatonin administration markedly attenuated paclitaxel-associated oxidative stress, as evidenced by reduced MDA levels and partial restoration of GSH content. These observations are in line with previous evidence supporting the broad cytoprotective actions of melatonin, which acts both as a direct free radical scavenger and as an indirect regulator of antioxidant defense systems [19,21]. Melatonin can access multiple intracellular compartments, enabling effective protection against oxidative and genotoxic stress [20]. In line with previous studies, melatonin treatment also suppressed caspase-3 activation, reduced 8-OHdG accumulation, and attenuated NF-κB-mediated inflammatory responses, thereby preserving testicular architecture and spermatogenic integrity [22,23]. Notably, melatonin administered alone was not associated with histological injury or increased lipid peroxidation; however, it was accompanied by a reduction in tissue GSH, suggesting modulation of the glutathione pool under basal conditions rather than overt oxidative damage. Therefore, interpretations of melatonin’s antioxidant action in this study rely primarily on its ability to counteract paclitaxel-driven oxidative burden.

Similarly, apigenin exerted robust protective effects against paclitaxel-induced testicular injury, as evidenced by reduced lipid peroxidation, improved antioxidant status, and suppression of apoptotic and inflammatory signaling pathways. These findings are consistent with previous studies demonstrating that apigenin modulates oxidative stress and apoptosis through multiple molecular targets, including inhibition of ROS generation and downregulation of pro-apoptotic signaling cascades [24,25]. Moreover, apigenin has been shown to inhibit NF-κB activation, supporting an additional anti-inflammatory mechanism contributing to its protective effects [26]. Notably, despite the reduction in GSH levels following apigenin administration alone, no histopathological damage or disruption of spermatogenesis was detected, as confirmed by preserved seminiferous tubule morphology and normal Johnsen scores. This dissociation between biochemical redox markers and tissue integrity suggests a context-dependent modulation of redox homeostasis without inducing structural or functional toxicity.

Although this pattern may be compatible with a hormetic-like response, such an interpretation cannot be conclusively established based on the present data alone. Alternatively, the observed decrease in GSH may reflect adaptive utilization of intracellular antioxidant reserves or metabolic regulation of glutathione turnover under basal conditions.

Importantly, under paclitaxel-induced oxidative stress, apigenin significantly improved both GSH levels and lipid peroxidation status, supporting its functional antioxidant role in pathological conditions. Collectively, these findings indicate that the antioxidant efficacy of apigenin is strongly influenced by the oxidative microenvironment and becomes biologically relevant primarily during chemotherapy-induced testicular injury.

Although both melatonin and apigenin exerted protective effects against paclitaxel-induced testicular injury, their modes of action appear to differ. Melatonin is a well-established direct free radical scavenger that enhances endogenous antioxidant defenses and stabilizes mitochondrial function, thereby rapidly restoring redox balance. In contrast, apigenin seems to modulate oxidative stress and inflammatory signaling in a context-dependent manner, as reflected by its differential effects on GSH levels under basal and stress conditions. In addition to its antioxidant activity, apigenin may exert its protective effects through regulation of NF-κB-mediated inflammatory pathways and apoptotic signaling. These findings suggest that while both agents are effective, melatonin may act primarily as a direct antioxidant, whereas apigenin may function as a redox modulator with broader regulatory effects.

A key strength of the present study is the direct comparative evaluation of melatonin and apigenin within the same experimental model. To our knowledge, this is the first study to directly compare the protective effects of these two bioactive compounds against paclitaxel-induced testicular injury under identical experimental conditions. These agents demonstrated comparable protective efficacy across biochemical, histological, and immunohistochemical parameters. Although both compounds exerted significant protective effects, subtle differences in their antioxidant and anti-inflammatory profiles may reflect distinct molecular mechanisms of action. Melatonin’s broad intracellular distribution and direct free radical scavenging capacity may confer superior protection against oxidative DNA damage, whereas apigenin’s modulation of signaling pathways may offer complementary anti-inflammatory benefits. These findings suggest that melatonin and apigenin may offer complementary protective mechanisms against chemotherapy-induced gonad toxicity.

The histopathological observations further supported the biochemical and immunohistochemical findings. Paclitaxel treatment resulted in severe degeneration of seminiferous tubules, germ cell loss, and significantly reduced Johnsen scores, indicating impaired spermatogenesis. In contrast, both melatonin and apigenin significantly preserved seminiferous tubule structure and improved Johnsen scores, indicating functional protection of the spermatogenic process. The correlation between biochemical markers and histological results highlights the biological significance of the observed protective effect.

The absence of a separate ethanol control group represents a potential methodological concern; however, its impact on the present findings is likely minimal. Intraperitoneal ethanol at concentrations ≤ 1% has consistently been reported to exert no measurable effects on testicular redox parameters or histological architecture in rodent models. Consistent with this literature, melatonin administration alone in the current study did not induce overt oxidative injury or morphological alterations in testicular tissue. While a confounding effect of ethanol cannot be completely excluded, the observed protective effects are more likely attributable to the pharmacological actions of melatonin.

Despite its strengths, the present study has certain limitations. Although apigenin and melatonin demonstrated improvements in oxidative stress parameters (MDA, GSH) as well as histological and immunohistochemical findings, functional sperm parameters such as sperm count, motility, and fertilization capacity were not evaluated. The relatively limited panel of oxidative stress and inflammatory markers represents an additional limitation, as key antioxidant enzymes and cytokines (e.g., SOD, CAT, TNF-α, IL-6) were not assessed and could provide deeper mechanistic insight. In addition, the absence of a separate ethanol (vehicle) control group represents a methodological limitation, although previous studies suggest that ethanol at low concentrations has minimal impact on testicular redox parameters and histological architecture. Furthermore, the potential combined effects of melatonin and apigenin were not examined, as the present design focused on the direct comparison of their individual protective profiles under identical experimental conditions. Future studies incorporating functional reproductive endpoints, dose–response analyses, a broader panel of oxidative and inflammatory markers, and combined treatment strategies are warranted to further clarify the therapeutic potential of melatonin and apigenin. These findings may also have translational relevance for the development of fertility-preserving strategies in patients undergoing chemotherapy.

## 5. Conclusions

The present study demonstrates that paclitaxel-induced testicular injury is associated with a coordinated increase in oxidative stress, apoptotic activity, inflammatory signaling, and structural degeneration of seminiferous tubules. Both melatonin and apigenin attenuated these alterations, as evidenced by improvements in redox balance, suppression of apoptotic and inflammatory markers, and preservation of testicular histoarchitecture.

Importantly, the differential effects observed under basal and oxidative stress conditions suggest that the antioxidant activity of these agents is context-dependent and becomes functionally relevant primarily in the presence of chemotherapy-induced oxidative injury.

While these findings provide mechanistic insight into the protective effects of melatonin and apigenin, the observed associations are correlative in nature and do not establish direct causal relationships. Furthermore, functional reproductive parameters were not assessed in the present study. Therefore, further studies incorporating functional fertility outcomes and targeted mechanistic approaches are required to better define the therapeutic potential of these compounds. These findings may have potential translational relevance and contribute to the development of novel fertility-preserving strategies in oncology.

## Figures and Tables

**Table 1:** Johnsen Testis Biopsy Score [29].

*Score*	
*1*	Germ cells and spermatogenesis are completely absent.
*2*	No germ cells, but Sertoli cells are present.
*3*	Only spermatogonia are present.
*4*	Only a few (<5) spermatozoa are present, and there are no spermatids or spermatozoa present.
*5*	There are no spermatozoa or spermatids, but a few or many spermatocytes are present.
*6*	There are no spermatozoa and only a few spermatids (<5–10) are present.
*7*	No spermatozoa present, but numerous spermatids are present.
*8*	Only a few spermatozoa (<5–10) are present.
*9*	Numerous spermatozoa are present, but the germinal epithelium is irregular and there is marked desquamation or closure of the lumen.
*10*	Spermatogenesis completed with numerous spermatozoa. The germinal epithelium is organized with uniform thickness and leaves a clear lumen.

**Table 2:** Biochemical Parameters.

Study Groups	Measured Parameters	
	MDA (TBARS) nmol/g Tissue	GSH µmol/g Tissue
DMSO	27.5 ± 1.12	3.55 ± 0.45 ^a***^
SF	20.08 ± 2.76 ^a**b*^	4.09 ± 0.39 ^a***^
Apigenin	22.4 ± 1.32 ^a*^	1.93 ± 0.42 ^a*b***c***^
Melatonin	19.6 ± 1.67 ^a**b*^	1.74 ± 0.15 ^b***c***d*^
Paclitaxel	28.7 ± 2.29	1.2 ± 0.09
Paclitaxel + Apigenin	17.2 ± 2.05 ^a***b**^	1.97 ± 0.04 ^a**b***c***^
Paclitaxel + Melatonin	18.4 ± 2.41 ^a**b**^	2.32 ± 0.11 ^a***b***c***^

Abbreviations: MDA, malondialdehyde; TBARS, Thiobarbituric acid reactive substances; GSH, reduced glutathione; DMSO, Dimethyl sulfoxide; SF, Saline. *: *p* < 0.05, **: *p* < 0.01, ***: *p* < 0.001, ^a^: Compared to the paclitaxel group, ^b^: Compared to the DMSO group, ^c^: Compared to the SF group, ^d^: Compared to the paclitaxel + melatonin group. One-way ANOVA/LSD Test.

**Table 3:** Johnsen Score Results (median-(25–75% interquartile range)).

Group	Johnsen Score
DMSO	9 (9–9)
SF	9 (8–9)
Apigenin	9 (8–9)
Melatonin	9 (8–9)
Paclitaxel	5 (4–6) **^a,b,c,d^**
Paclitaxel + Apigenin	8 (8–9) **^a,e^**
Paclitaxel + Melatonin	8 (7–8) **^a,^^b,c,d,e^**

**^a^** *p* < 0.05 Compared to the DMSO group, **^b^** *p* < 0.05 Compared to the SF group Group, **^c^** *p* < 0.05 Compared to the apigenin group, **^d^** *p* < 0.05 Compared to the melatonin group, **^e^** *p* < 0.05 Compared to the paclitaxel group, Kruskal–Wallis/Dunn test.

**Table 4:** Immunohistochemical Positivity Score Results (median-(25th–75th percentile range)).

Group	Caspase-3 Positivity Score	8-OHdG Positivity Score	NF-κB/p65 Positivity Score
DMSO	0 (0–0)	0 (0–0)	0 (0–0)
SF	0 (0–0)	0 (0–0)	0 (0–0)
Apigenin	0 (0–0)	0 (0–0)	0 (0–0)
Melatonin	0 (0–0)	0 (0–1)	0 (0–1)
Paclitaxel	3 (2–3) ^a,b,c,d^	3 (2–3) ^a,b,c,d^	2 (2–2) ^a,b,c,d^
Paclitaxel + Apigenin	1 (0–1) ^a,b,c,d,e^	1 (0–1) ^a,b,c,d,e^	0.5 (0–1) ^a,b,c,d,e^
Paclitaxel + Melatonin	1 (1–1) ^a,b,c,d,e^	1 (1–1) ^a,b,c,d,e^	1 (0–1) ^a,b,c,d,e^

^a^ *p* < 0.05 Compared to the DMSO group, ^b^ *p* < 0.05 Compared to the SF group, ^c^ *p* < 0.05 Compared to the apigenin group, ^d^ *p* < 0.05 Compared to the melatonin group, ^e^ *p* < 0.05 Compared to the paclitaxel group, Kruskal–Wallis/Dunn test.

## Data Availability

The datasets generated during the current study are available from the corresponding author upon reasonable request.

## Abbreviations

The following abbreviations are used in this manuscript:

8-OHdG	8-hydroxy-2′-deoxyguanosine
DAB	3,3′-diaminobenzidine
DMSO	Dimethyl Sulfoxide
DNA	Deoxyribonucleic Acid
DTNB	5,5′-dithiobis-(2-nitrobenzoic acid)
GSH	Reduced glutathione
H&E	Hematoxylin and eosin
i.p.	Intraperitoneal
MDA	Malondialdehyde
NaCl	Sodium chloride
NF-κB	Nuclear factor kappa B
ROS	Reactive Oxygen Species
SF	Saline

## References

[1] Abbas Z., Rehman S., Abbas Z., Rehman S. (2018). An Overview of Cancer Treatment Modalities. Neoplasm.

[2] Abd-Elrazek A.M., El-dash H.A., Said N.I. (2020). The role of propolis against paclitaxel-induced oligospermia, sperm abnormality, oxidative stress and DNA damage in testes of male rats. Andrologia.

[3] Aboelwafa H.R., Ramadan R.A., El-Kott A.F., Abdelhamid F.M. (2022). The protective effect of melatonin supplementation against taxol-induced testicular cytotoxicity in adult rats. Braz. J. Med. Biol. Res..

[4] Asma M., Fouzia T., Lazhari T., Khireddine O. (2020). Protective effects of apigenin against Bisphenol A-induced testis toxicity in Wistar rats through modulating hepatic biochemical biomarkers and histological changes. Comp. Clin. Pathol..

[5] Baş E., Nazıroğlu M. (2019). Treatment with melatonin and selenium attenuates docetaxel-induced apoptosis and oxidative injury in kidney and testes of mice. Andrologia.

[6] Borovskaya T.G., Goldberg V.E., Rumpel O.A., Pahomova A.V., Perova A.V., Goldberg E.D. (2009). The Rat Spermatogenesis after Injection of Paclitaxel (Antitumor Agent). Bull. Exp. Biol. Med..

[7] Bray F., Laversanne M., Sung H., Ferlay J., Siegel R.L., Soerjomataram I., Ahmedin J. (2024). Global cancer statistics 2022: GLOBOCAN estimates of incidence and mortality worldwide for 36 cancers in 185 countries. CA Cancer J. Clin..

[8] Bray F., Ferlay J., Soerjomataram I., Siegel R.L., Torre L.A., Jemal A. (2018). Global cancer statistics 2018: GLOBOCAN estimates of incidence and mortality worldwide for 36 cancers in 185 countries. CA Cancer J. Clin..

[9] Delkhoshe-Kasmaie F., Malekinejad H., Khoramjouy M., Rezaei-Golmisheh A., Janbaze-Acyabar H. (2014). Royal jelly protects from taxol-induced testicular damages via improvement of antioxidant status and up-regulation of E2f1. Syst. Biol. Reprod. Med..

[10] du Sert N.P., Hurst V., Ahluwalia A., Alam S., Avey M.T., Baker M., Browne W.J., Clark A., Cuthill I.C., Dirnagl U. (2020). The ARRIVE Guidelines 2.0: Updated Guidelines for Reporting Animal Research. PLoS Biol..

[11] Ellman G.L. (1959). Tissue sulfhydryl groups. Arch. Biochem. Biophys..

[12] Johnsen S.G. (1970). Testicular Biopsy Score Count—A Method for Registration of Spermatogenesis in Human Testes: Normal Values and Results in 335 Hypogonadal Males. Horm. Res..

[13] Kampan N.C., Madondo M.T., McNally O.M., Quinn M., Plebanski M. (2015). Paclitaxel and Its Evolving Role in the Management of Ovarian Cancer. BioMed Res. Int..

[14] Marupudi N.I., Han J.E., Li K.W., Renard V.M., Tyler B.M., Brem H. (2007). Paclitaxel: A review of adverse toxicities and novel delivery strategies. Expert Opin. Drug Saf..

[15] Mayo J.C., Sainz R.M., Tan D.X., Hardeland R., Leon J., Rodriguez C., Reiter R.J. (2005). Anti-inflammatory actions of melatonin and its metabolites, N1-acetyl-N2-formyl-5-methoxykynuramine (AFMK) and N1-acetyl-5-methoxykynuramine (AMK), in macrophages. J. Neuroimmunol..

[16] Meerum Terwogt J.M., Nuijen B., Ten Bokkel Huinink W.W., Beijnen J.H. (1997). Alternative formulations of paclitaxel. Cancer Treat. Rev..

[17] Miles A., Philbrick D.R.S. (1988). Melatonin and psychiatry. Biol. Psychiatry.

[18] Nisari M., Kaymak E., Ertekin T., Ceylan D., Inanc N., Ozdamar S. (2019). Effects of Paclitaxel on Lipid Peroxidation and Antioxidant Enzymes in Tissues of Mice Bearing Ehrlich Solid Tumor. Eurasian J. Med. Investig..

[19] Ohkawa H., Ohishi N., Yagi K. (1979). Assay for lipid peroxides in animal tissues by thiobarbituric acid reaction. Anal. Biochem..

[20] Porter A.G., Jänicke R.U. (1999). Emerging roles of caspase-3 in apoptosis. Cell Death Differ..

[21] Prince Vijeya Singh J., Selvendiran K., Mumtaz Banu S., Padmavathi R., Sakthisekaran D. (2004). Protective role of Apigenin on the status of lipid peroxidation and antioxidant defense against hepatocarcinogenesis in Wistar albino rats. Phytomedicine.

[22] Reiter R.J. (1991). Pineal melatonin: Cell biology of its synthesis and of its physiological interactions. Endocr. Rev..

[23] Rodriguez C., Mayo J.C., Sainz R.M., Antolín I., Herrera F., Martín V., Reiter R.J. (2004). Regulation of antioxidant enzymes: A significant role for melatonin. J. Pineal Res..

[24] Salehi B., Venditti A., Sharifi-Rad M., Kręgiel D., Sharifi-Rad J., Durazzo A., Lucarini M., Santini A., Souto E.B., Novellino E. (2019). The Therapeutic Potential of Apigenin. Int. J. Mol. Sci..

[25] Serizawa K.I., Yogo K., Aizawa K., Tashiro Y., Takahari Y., Sekine K., Suzuki T., Ishizuka N., Ishida H. (2012). Paclitaxel-induced endothelial dysfunction in living rats is prevented by nicorandil via reduction of oxidative stress. J. Pharmacol. Sci..

[26] Singla A.K., Garg A., Aggarwal D. (2002). Paclitaxel and its formulations. Int. J. Pharm..

[27] Snigdha S., Smith E.D., Prieto G.A., Cotman C.W. (2012). Caspase-3 activation as a bifurcation point between plasticity and cell death. Neurosci. Bull..

[28] Sönmez M., Ozdemir Ş., Guzel M., Kaymak E. (2017). The ameliorative effects of vinpocetine on apoptosis and HSP-70 expression in testicular torsion in rats. Biotech. Histochem..

[29] Wang Z.X., Teng Z., Wang Z.L., Song Z., Zhu P., Li N., Zhang Y.S., Liu X.X., Liu F.J. (2022). Melatonin ameliorates paclitaxel-induced mice spermatogenesis and fertility defects. J. Cell. Mol. Med..

